# Current and Future Photography Techniques in Aesthetic Surgery

**DOI:** 10.1093/asjof/ojab050

**Published:** 2021-11-29

**Authors:** Shyon Parsa, Berkay Basagaoglu, Kate Mackley, Patricia Aitson, Jeffrey Kenkel, Bardia Amirlak

**Affiliations:** Department of Plastic Surgery, UT Southwestern Medical Center, Dallas, TX, USA

## Abstract

**Background:**

The rapidly increasing modalities and mediums of clinical photography, use of 3-dimensional (3D) and 4-dimensional (4D) patient modeling, and widening implementation of cloud-based storage and artificial intelligence (AI) call for an overview of various methods currently in use as well as future considerations in the field.

**Objectives:**

Through a close look at the methods used in aesthetic surgery photography, clinicians will be able to select the modality best suited to their practice and goals.

**Methods:**

Review and discussion of current data pertaining to: 2-dimensional (2D) and 3D clinical photography, current photography software, augmented reality reconstruction, AI photography, and cloud-based storage.

**Results:**

Important considerations for current image capture include a device with a gridded viewing screen and high megapixel resolution, a tripod with leveling base, studio lighting with dual-sourced light, standardized matte finish background, and consistency in patient orientation. Currently, 3D and 4D photography devices offer advantages such as improved communication to the patient on outcome expectation and better quality of patient service and safety. AI may contribute to post-capture processing and 3D printing of postoperative outcomes. Current smartphones distort patient perceptions about their appearance and should be used cautiously in an aesthetic surgery setting. Cloud-based storage provides flexibility, cost, and ease of service while remaining vulnerable to data breaches.

**Conclusions:**

While there are advancements to be made in the physical equipment and preparation for the photograph, the future of clinical photography will be heavily influenced by innovations in software and 3D and 4D modeling of outcomes.

Clinical photography has been a mainstay in aesthetic surgery since the 1900s.^1^ The importance of clinical photography to a field as visual as plastic surgery was immediately clear, and 2-dimensional (2D) still photographs of patients preoperatively and postoperatively became a mainstay in clinical practice. As the need for improved quality and consistency of the photographs has been elucidated, newer camera systems and a streamlined, standardized clinical setup are becoming standard in aesthetic surgery.

Advances in the image acquisition and processing methodology have helped create a future in photography which will be markedly different from the methods of the recent past. This 2-part review aims to discuss both the current and future considerations in photography methods and software.

## METHODS

The authors reviewed current and previous articles using a PubMed search with key search terms such as 2D clinical photography, 3D clinical photography, current photography software, augmented reality reconstruction, artificial intelligence (AI) photography, mobile data storage, and cloud-based storage. Informed consent was obtained from all patients involved in the study, and ethical review and approval were waived for this study, due to the nature of the study as a review of existing literature. Experiments were not performed on human or animal patients.

## RESULTS

### Current Photography Methods

#### Imaging Device

Photography in aesthetic plastic surgery relies on digital single-lens reflex cameras (DSLRs) or, more recently, mirrorless cameras.^2^ The difference between the 2 types of camera is the addition of a mirror in the DSLR that allows the user to see the exact representation of the photograph to be taken as opposed to the digitally reconstructed image that the mirrorless camera provides. The rear crystal display that both camera types utilize allows the user to review images before taking the shot.^3^ Additionally, a gridded viewing screen can properly frame and center the patient’s body part of interest.^3^ Mirrorless cameras offer the additional advantages of smaller size and less weight, and image stabilization that is not limited to solely the lens, but both the lens and the image sensor.^4^

Resolution of 5 megapixels or higher is the bare minimum for publication (2500 by 1800 pixels producing a 6- by 9-inch image), a value surpassed easily by current cameras.^5^ As reference, current smartphones have resolutions from 8 to 12 megapixels and beyond. A 12-megapixel DSLR camera (4200 by 2800 pixels image producing an 8 by 12 image) is ideal for visual inspection of the edges of the pictures and improved zooming in while retaining definition. Currently, a range of resolutions are in use, from clinical practices using 25 megapixels to cameras capable of shooting at 45 megapixels and beyond.

**Figure 1.**
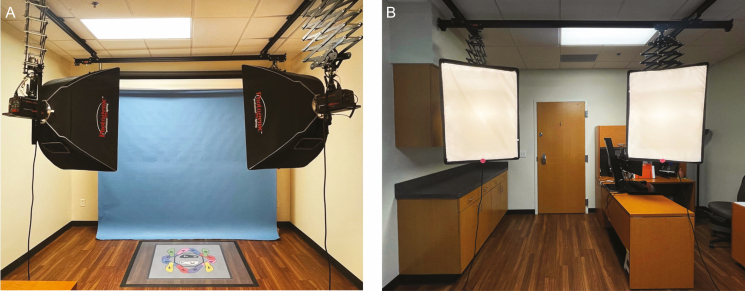
Studio lighting positioned at 45-degree angles from patient, displayed from the perspective of both the (A) photographer and (B) patient.

While the physical properties of the camera are important, the accessories can lead to marked improvements in consistency and reproducibility in the image. Current practices utilize a tripod with leveling base to ensure that the photograph does not deviate due to difficult to discern user-based changes (Figure 1). Even a couple of degrees of tilt can lead to a different perception of aesthetic outcomes from both the patient and clinician. As an example, a slight posterior tilt of the head in a patient who underwent a blepharoplasty alters eye shape, the tilt of the intercanthal axis, and conceals scleral show, potentially distorting the postoperative image.^6^ A prime lens creates a reproducible focal length from patient to camera as opposed to a zoom lens.^7,8^ A prime lens shoots at a fixed focal distance, irrespective of the distance between camera and patient. A zoom lens can be adjusted by the photographer to properly focus the patient but leads to human-introduced error. Thus, the tripod and prime lens allow for minimized user destabilization and create consistency between sets of images (ie, pre vs post).

#### Lighting

Lighting is an important consideration to ensure consistency and uniformity of clinical photographs and is of special importance in aesthetic clinical photography. The use of a single on-camera flash produces harsh lighting. Due to the focal nature of the source, this also creates an uneven spread of the light on the patient.^3^ An alternative for a more even distribution of light is the ring flash. The central issue with this method is the washing out of patient skin tone. This method also flattens the patient image by eliminating shadows on the patient, thereby creating a loss of discernable landmarks such as the shadowing around the nasolabial fold. A third option is a studio lighting setup that has 2 sources of light arranged at a 45-degree angle in front of the patient (Figure 1). The studio lighting highlights the ideal scenario, and should this not be available, the previously mentioned practical and cost-effective lighting solutions will suffice as well. This method creates shadows that are useful to both the clinician and patient as progress markers and also creates shadows symmetrically on the patient’s area of interest. The use of an umbrella improves upon this method by diffusing light to create an even spread on the patient. Of note, an umbrella creates wash out so a focal light source such as a single flash or cross-polarized light should be used in conjunction with the umbrella to create the desired contour and shadowing in certain circumstances. Depending on the nature of a particular case, the intensity and angle of lighting can be adjusted. For example, a downlight that intentionally moves the light above the patient will highlight aging features and scars by creating shadows.

Aperture size is determined by the level of illumination present. Sufficient illumination would allow for the use of a narrow aperture which would create a larger focal plane. Lower illumination would require a wide aperture and this limits the plane in focus to a small area on the patient (ie, the nose would be in focus but the periorbital area remains blurry).

A final consideration is a light temperature and the effect on aesthetic appearance. The temperature of clinical light can range from 3000 Kelvin to approximately 5500 Kelvin.^9^ The use of “warm” light can soften the look of the patient at the expense of distorting the skin tone. “Cold” light, generally whiter and approaching the higher Kelvin temperatures, decreases the appearance of wrinkles but casts a harsher tone on the patient. Generally in aesthetic plastic surgery, the ideal light temperature would be near the higher ranges, 5000 to 5500 Kelvin, to keep the photograph as accurate as possible with minimal distortion.

#### Background

Background color can influence the perception of skin tone and brightness by an observer. For clinical photographs, the recommendation is a studio blue, black, or white gray background.^10^ The standard white background produces vivid shadowing and a black background decreases the 3-dimensional (3D) quality of the photograph.^11^ Within the clinical setting, a fixed spot for the background allows for internal consistency.^2^ The material of the background should be nonreflective material with a matte finish.

#### Patient Considerations

There are a variety of possible patient considerations that must be controlled for photographic consistency across both time and clinics. The patient should remove any accessories such as jewelry, hats, masks, and glasses for the photographs. If possible, patients should not be wearing any makeup for the photographs. Hair should be pulled back with elastics or headbands. The patient’s facial position should remain consistent between preoperative and postoperative photographs.

**Figure 2.**
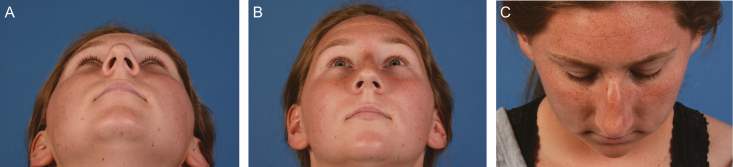
Preoperative photographs of a 23-year-old female patient in (A) worm’s eye view, (B) modified 45-degree worm’s eye view, and (C) bird’s eye view.

Positional changes of the patient allow for a more complete picture of patient outcomes. Generally, there are 5 proposed views for aesthetic photography, anterior, bilateral oblique, and lateral views. Of note, there are procedures where posterior views are appropriate and patient positioning is ultimately at the discretion of the clinician. For most facial aesthetic surgery, defining the Frankfort plane for each patient and ensuring this plane is parallel to the ground allow for standardization of clinical photographs.^12-14^ The imaginary line originating from the top of the tragus to the infraorbital rim defines the Frankfort plane superiorly and inferiorly, and the transverse extension of this line creates the plane.^12^ There have been proposals to improve the alignment of the face in photographs using the NETWORK line, which extends from the superior surface of the pinna to the lateral canthus.^10^ By tilting the face more downward than the Frankfort line, this allows for a more comprehensive look at submental fat. Different cosmetic operations do introduce additional or different patient views. As an example, an otoplasty will require a posterior and lateral photograph of the ear. A rhinoplasty has an inferior and superior views, colloquially called the Worm’s and Bird’s eye views, respectively.^15^ (Figure 2)

### History of 2D Photography Software

Two-dimensional photography has evolved due to software advances in digitalization, storability, and image enhancement. Early progress in the field of photographic software came about in the late 1990s, with the advent of digital photography.^16^ This allowed for not only taking photographs with a digital camera but also storing and archiving the information in an organized and more easily accessible manner. Post digital revolution, advancements in software in the 2000s and 2010s began to shape the landscape commonplace in clinical photography today. Software allowed for immediate image enhancement, including resolution enhancement, motion correction, simple digital zoom, and brightness adjustment at the time of image capture.^17^ Various software specific to aesthetic surgery introduced in the 2010s provided an automatic objective measure of symmetry and morphological changes preoperatively and postoperatively.^18^ Different permutations of this software have been created, and by calculating the distances, areas, and changes in the patient’s features, they allow for a more rapid analysis when compared with previous methods such as ImageJ (National Institute of Health, Bethesda, MD).

While still photography remains widely used in clinical practice today, recent advances in software focus on 3D models, a route many consider to be the present and future of the subfield.^18,19^

### Current 3D Photography

The advent of 3D photography brought improvements to the previous 2D imaging by providing additional information about contour and shape.^19^ The validity of 3D stereophotogrammetry, the method of acquiring the images by combining photographs from various angles, has been studied for various systems.^20,21^ Although other methods of imaging exist, such as computed tomography renderings, ultrasonography, and laser scanning, this review will focus on software and devices that utilize 3D stereophotogrammetry as this technology has a reduced risk to patients and is widely utilized by plastic surgery practices.

There are various methods to construct the 3D model using a stereophotogrammetry device, a method that arose in the late 1970s and early 1980s.^22^ Initial designs began with the patient being photographed from at least 2 different planes with image construction software then aligning the data for a manipulatable 3D rendered model.^23^ The software defines a list of points originating from each imaging device that correlate to a location in the 3D space, and the recognition of similar points across individual images allows for the reconstruction.^24^ The method of “point cloud construction” was later improved through the combination of multiple digital photographs and a software algorithm that scans patches of each area for corresponding points. This method, labeled photograph-based scanning, results in a collection of 3D, increasingly dense point clouds.^24^ In contrast to 2D medical photograph studios, these camera systems are easily placed in a regular clinic room with no need for additional specialized lighting.

Post-acquisition, there is software that can be of further use to the clinician based on specific needs. Custom software can align multiple 3D datasets across different time points, allowing the clinician to easily observe longitudinal changes and the healing process in the structures postoperatively.^25^ In addition to longitudinal change, there is a need to identify the specific aesthetic regions on sequential images in a postoperative patient for topographic measurement and volume analysis. These features require fixed landmarks, and while this may be manually performed in certain features with reproducible landmarks, mobile target areas such as the cheeks or jowls prove more difficult to measure, especially when considering marked volumetric differences that could happen due to expression change.^26^ Recent advances in software have allowed for the creation of a personalized template that tracks changes in facial landmarks.^27^ By first creating a standard aesthetic template, the use of a nonrigid transformation (coherent point drift) will morph the patient’s 3D model to fit the standard. Volume measurements of the regions needed by the clinician can be ascertained with ease. Many of the current 3D photography devices utilize similar software to provide objective data to the clinician.

There are numerous 3D systems currently used in aesthetic surgery including the Vectra XT, H2 (Canfield Scientific Inc., Fairfield, NJ), Crisalix VR 4D systems (Crisalix SA, Lausanne, Switzerland), 3dMD (3dMD Inc, Atlanta, GA), and Morpheus 3D (Morpheus 3D Co., Ltd, Seongnam, South Korea) (Table 1).

**Table 1.** Comparison of 3-Dimensional (3D) Modeling Systems

Device	Size	Advantages	Disadvantages
3D Vectra XT	42 cm (H) by 183 cm (W)	Validated through multiple studies Facial skin analysis software Tools available for longitudinal outcome tracking	Large device size Single static 3D model without augmentation
Vectra H2	32 cm (H) by 18 cm (W)	Portability Ease of use Intellistage features allow for 360-degree imaging Facial skin analysis software Tools available for longitudinal outcome tracking	Fewer studies validating use Single static 3D model without augmentation
Crisalix VR 4D	Portable device add-on	Augmented reality offers novel patient perspective Variety of cosmetic surgical outcomes modeled	Basic skin analysis software Utility limited to preoperative modeling
ILLUSIO Pro	Portable device add-on	Augmented reality offers novel patient perspective Ease of use Requires only portable device and software	Basic skin analysis software Outcome modeling focused only on breast Utility limited to preoperative modeling

3D, three-dimensional; 4D, four-dimensional; H, height; W, width.

**Figure 3.**
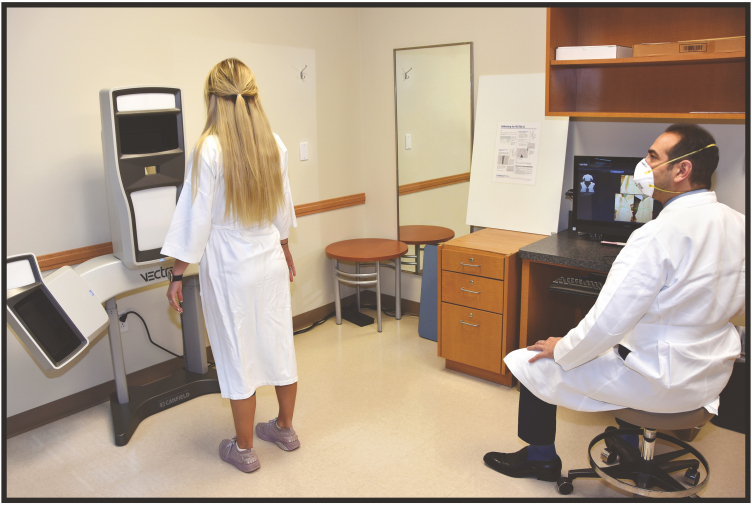
Clinical setup of 3D Vectra XT and device with a 25-year-old female patient. 3D, three-dimensional.

The 3D Vectra XT is a 42 cm by 183 cm multiple camera system that saves images to a local computer connected to the device. The reconstruction is uploaded to an interface that allows the clinician to assess metrics such as symmetry, proportions, and angles of interest (Video 1). After selecting the specific cosmetic operation to be performed, the software can project a rendering of a potential surgical outcome after the clinician and patient decide on the ideal properties of the final reconstruction. On follow-up examinations, the Vectra system is capable of marking surface irregularities and volume changes due to localized swelling. The Vectra XT 3D reconstructions have value as a measure of aesthetic outcome of the facial surface.^28,29^ The Vectra H2 system is also capable of face and body imaging, but in a smaller and portable device. The device has a couple of differences from the Vectra XT. The XT allows the overlaying of a simulated 3D projection of possible surgical outcomes visualized on the patient’s current preoperative image. The H2 has an “Intellistage” feature, which allows for a controlled 360-degree turn to obtain images from all angles of the patient body and face. The H2 also has a facial skin analysis feature that provides objective analysis of spots, wrinkles, brown spots, red areas, pores, and the texture of the patient’s face. A few existing studies have used the Vectra H2 system as an imaging and analysis device, but the novelty of the H2 creates an opportunity for further research^30,31^ (Figure 3).

**Figure 4.**
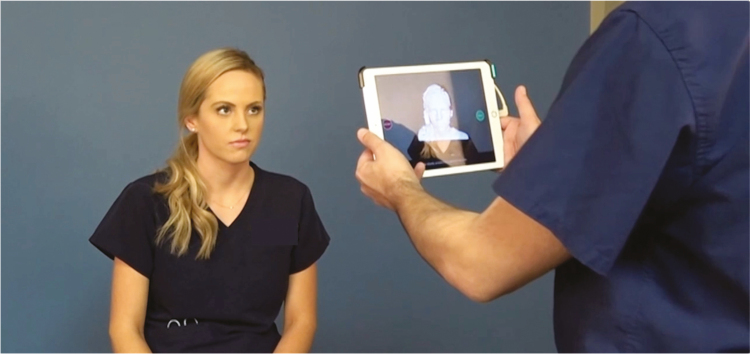
Clinical use of Crisalix VR 4D device using a portable electronic device with a 26-year-old female patient. 4D, four-dimensional.

The Crisalix VR 4D builds on existing 3D modeling by augmenting the 3D reconstruction on the patient themselves, a more integrated experience (Video 2). This form of augmented reality uses a device attachment on an iPad to first create the 3D image and then project a potential postoperative outcome directly on the patient. The technology uses either a mirror or more recently glasses as the medium between the real world and reconstruction. The patient can now move beyond a freestanding model and understand at a more intimate level what the aesthetic surgery will look and feel like postoperatively. The Crisalix device is currently capable of modeling outcomes of facial aesthetic surgery, including rhinoplasty, facelifts, and lip augmentation; breast cosmetic procedures, such as augmentation, reduction, or implant revision; and cosmetic body procedures that include liposuction and body contouring. The benefits of the imaging are improved communication to the patient and education of clinicians, which improves the quality of patient service and safety^32,33^ (Figure 4).

In addition to device-based imaging technology, the ILLUSIO Pro (Illusio Imaging, San Clemente, CA) is a software add-on for an iPad that allows the patient to view postoperative outcomes of breast augmentation, reduction, or lift with real-time adjustments in clinic. The patient can adjust metrics such as size, roundness, sag, lift, and cleavage to their liking based on the software reconstruction, which will allow the clinician a deeper understanding of the patient’s goals. While various studies have examined the benefits of augmented reality and patient perception of outcome, none have specifically assessed the ILLUSIO device at this time.

These devices help aid the patient educationally to better understand the outcomes that may be achieved with surgery. That being said, the patients must be educated on the limitations of existing technology to temper expectations and disclose variations that occur during cosmetic procedures. In no way should the patients interpret these simulations as a guarantee of their surgical outcome.

## Discussion

### Future Considerations in Imaging Methods

Clinical photography has been a cornerstone in cosmetic plastic surgery for decades. As photographic equipment advances, there will be a similar advancement in the devices used in clinical imaging. As current imaging devices far exceed the megapixel threshold for detailed clinical photography, the true advances in this aspect of the photographic process may occur in the software, as discussed later in this paper.

A consideration for future changes in the illumination of aesthetic clinical photographs is the rapidly growing field of AI. AI mimics human intelligence by implementing specific algorithms, artificial neural networks, akin to human decision making, to predict and automate tasks, including facial analysis and surgical planning. Machine learning is a subcategory of AI, and deep learning is a subcategory of machine learning, that differs based on the number of data points and the ability of the algorithm to adapt to increasing datapoints.^34^ While current illumination methods may rely on user adjustment of the lighting settings, advances in AI would create “Smart Lighting” of clinical photographs. A clinician would feed the software with the outcome of interest and the AI would output properly illuminated photographs. Consistency remains key in clinical photography, and through AI-adjusted lighting, images may be replicated at a level not possible using the naked eye. Such advances are being explored in the sphere of biological imagery and consumer electronics, and thus the transition to clinical photography may not be far behind.^35^ Using AI for predicting cosmetic procedure outcomes has proven that the practice is positively perceived by patients, both in confidence scores and in satisfaction.^36,37^ This trend will very likely continue with advancements in AI algorithms providing improved outcome prediction in a variety of cosmetic procedures.

While currently in clinical practice the extent of 3D and 4D modeling is a virtual reconstruction of the patient, the use of physical 3D models has also begun to be introduced into cosmetic surgery. Three-dimensional printing of the postoperative outcome of rhinoplasty was one of the earliest uses of the method.^38^ Patients expressed high satisfaction with the model, citing increased confidence in the procedure goals and higher helpfulness when compared with solely using the computer 3D simulation.^39^ As 3D printing becomes increasingly cost effective, there is evidence to suggest the widespread incorporation of 3D modeling into other cosmetic procedure consultations in the future.

Finally, there is a push to transition from photography to video to obtain more clinically comprehensive records and allow for tracking of changes in patient movement preoperatively and postoperatively.^40^ Videography provides details that static images are unable to provide such as changes in the patient’s anatomy when in motion, the effect of patient body position such as sitting or smiling on various body parts, or a more clinically comprehensive, permanent record of postoperative goals the patient has preoperatively.

### Smartphones, Video, and Data Storage

Rapid advancements in software have led to mobile applications which can create 3D models of patients using just the portable device. While some have touted mobile devices as the future of imaging in cosmetic surgery, there are nuances to consider. There is a clear discrepancy between the power of predictive modeling on a phone vs a dedicated device both in image resolution and computing power. Additionally, there are differences in the quality of cameras, software capabilities, etc. between the various brands of smartphones. Standardized imaging modalities would dissipate this issue. Patients have also increasingly been using front-facing smartphone camera photographs (a “selfie”) during discussions of goals with aesthetic surgeons. Front-facing cameras introduce distortion in the final photograph, especially in nasal length, and should be used with caution in this setting.^41^

Video may present data storage issues, a growing concern considering the volume of patient records with the passage of time. A high-definition photograph can necessitate 5 to 10 MBs per image depending on camera resolution, whereas an aesthetic patient video series shot in 1080p with a 24 frame per second (fps) rate will consume approximately 70 to 100 MBs. Using lower resolution settings for either of these recording or imaging modes will save data space, but considerations for later publication or maintaining the quality of care should be balanced against this tradeoff.

The growing database of patient images has created the necessity for a low-cost and rapidly accessible storage medium: Cloud-based storage.^42^ “Cloud storage” is defined by the access of data storage through the internet along with the capacity to read and write these data remotely. In addition to lower cost and ease of access, cloud storage allows for simple image sharing with other providers, flexibility in storage capability with no hard limit set by hardware, and easy service when issues arise due to a technician needing to be physically present for repairs.^43^ However, the remote aspect of cloud storage introduces the vulnerability to patient privacy and security breaches which would violate the Health Insurance Portability and Accountability Act of 1996 (HIPAA).^44^ Data encryption must ensure that only authorized users, those with approved access to clinical documentation, are viewing the patient images.^43^ Even by ensuring user access is limited, a data breach would be more harmful considering the volume of patient images that could be stored. As an example, in January 2021, data from the 20/20 Hearing Care Network, stored on Amazon Web Services, were hacked into and patient information was downloaded and deleted before the breach could be contained.^45^ Considering the pros and cons of remote storage will be an important balance to strike moving forward. Generally, with improvements in cloud security and certifications required of the web-based service to show compliance with federal health storage guidelines, cloud-based storage remains a viable and growing option for the storage of patient photographs and videos.

## Conclusions

Clinical photography is an important tool in cosmetic surgery. While there are advancements to be made in the physical equipment and preparation for the photograph, the future of clinical photography will be heavily influenced by innovations in software. Three-dimensional imaging has provided clinicians with insight into the structure of the patient body to an extent not seen previously, and continued advancements in AI modeling and more insightful imaging software will continue to drive future clinical decisions.
